# Cavernous Hemangioma of the Tongue Base: A Rare Case

**DOI:** 10.7759/cureus.69759

**Published:** 2024-09-19

**Authors:** Fathmath Shana Mohamed, Bee-See Goh

**Affiliations:** 1 Otolaryngology - Head and Neck Surgery, Universiti Kebangsaan Malaysia, Kuala Lumpur, MYS

**Keywords:** base of the tongue, cavernous hemangioma, head and neck neoplasms, hemangioma, vascular anomalies

## Abstract

Vascular anomalies encompass a range of conditions affecting blood vessel development, categorized as tumors or malformations. Hemangiomas, the most common vascular tumors, involve abnormal endothelial cell proliferation, particularly in hemangiomas, which are prevalent benign tumors arising from mesenchymal tissue in the head and neck. They manifest as capillary, cavernous, or mixed types, affecting areas like the tongue and lips. Hemangiomas of the tongue base are notably rare, emphasizing the complexity of diagnosis and management due to their uncommon occurrence and potential for complications like bleeding. This report highlights a case of cavernous hemangioma of the tongue base, underscoring diagnostic challenges and management considerations.

A Malay man in his late 30s, a nonsmoker and nondrinker, presented with a year-long history of intermittent globus sensation without associated symptoms like odynophagia, dysphagia, intraoral bleeding, or neck swelling. Flexible nasopharyngolaryngoscopy revealed a lobulated bluish mass at the right base of the tongue, prompting a provisional diagnosis of hemangioma. Contrast-enhanced CT suggested an irregular lesion with calcification, leading to MRI confirmation of a well-defined, non-muscle-invasive lesion that favored venolymphatic malformation rather than hemangioma. However, it was confirmed histologically as cavernous hemangioma after excision, where intraoperative findings aligned with initial preoperative clinical assessments.

## Introduction

Vascular anomalies encompass a diverse array of conditions characterized by aberrations in blood vessel development and function, which can be broadly classified as vascular tumors or vascular malformations. Vascular tumors, which may present at birth or arise later in life, are characterized by abnormal cellular proliferation, predominantly involving endothelial cells [1]. Among vascular tumors, hemangiomas stand out as the most prevalent type, characterized by their distinctive proliferation of blood vessels [2].

Hemangioma, originating from the Greek roots for "blood vessel tumor" (*haima* - blood, *angeion* - vessel, *oma* - tumor), denotes a benign proliferation characterized by the dilation of blood vessels. Constituting approximately 7% of all benign tumors in the maxillofacial region, hemangiomas arise from mesenchymal tissue and are classified as benign congenital hamartomas. They are further categorized into capillary, cavernous, or mixed types based on vascularization patterns. These growths commonly manifest in diverse head and neck areas, including the tongue, lips, buccal mucosa, gingiva, palatal mucosa, salivary glands, alveolar ridge, and jaw bones [3].

While lingual hemangiomas are relatively common, those localized at the tongue base are exceedingly rare occurrences [4]. The rarity of hemangiomas of the tongue base underscores the complexity and variation within this condition, demanding careful consideration in diagnosis and management. Special attention should be given to hemangiomas in the tongue due to their vulnerability to minor injuries leading to bleeding and ulceration.

We intend to highlight the diagnosis of a case of cavernous hemangioma involving the tongue base.

## Case presentation

A Malay man in his late 30s, a nonsmoker and nondrinker, presented with intermittent globus sensation persisting for about a year. He did not report odynophagia, dysphagia, intraoral bleeding, neck swelling, or symptoms suggestive of upper airway obstruction. There were no associated constitutional symptoms, and he had no family history of head and neck malignancy. Physical examination revealed no neck swelling or other lesions on the body.

Flexible nasopharyngolaryngoscopy revealed a lobulated bluish mass at the right base of the tongue, with the lingual surface of the epiglottis appearing normal and bilateral vocal cords mobile and no pooling of saliva (Video 1). Our provisional diagnosis was hemangioma of the right base of the tongue.

**Video 1:** Flexible nasopharyngolaryngoscopy revealed a lobulated bluish mass at the right base of the tongue, with the lingual surface of the epiglottis appearing normal and bilateral vocal cords mobile, and no pooling of saliva

**Figure 1:**
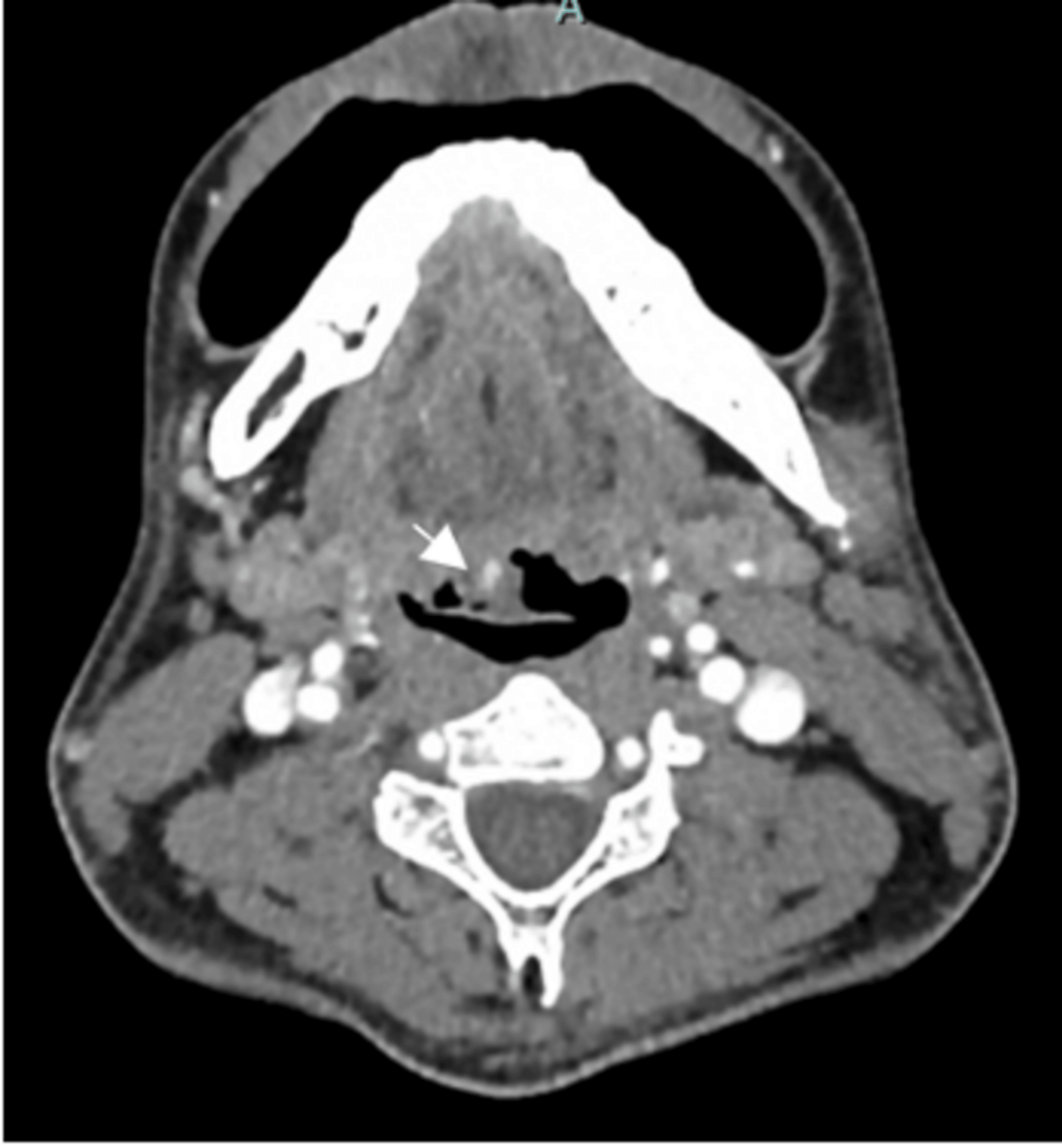
Axial CECT neck showing irregular exophytic enhancing lesion at the right base of the tongue (white arrow). There is hyperdensity at the right base of the tongue to suggest calcification. It abuts the epiglottis. CECT: contrast-enhanced computed tomography

**Figure 2:**
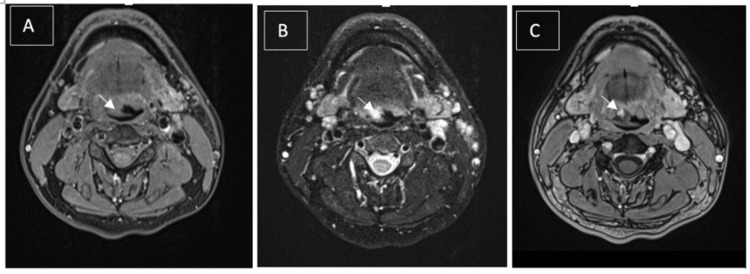
(A) T1-weighted axial MRI neck, (B) T2-weighted axial MRI neck, and (C) T1-weighted axial MRI neck (post-contrast) (A, B, C): A well-defined lesion is observed at the right base of the tongue, appearing homogeneously isointense on T1 and hyperintense on T2. It does not involve the extrinsic muscles of the tongue but abuts the vallecula. Post-contrast images show incomplete enhancement of the lesion at the right tongue base, with no arterial supply to the lesion. MRI: magnetic resonance imaging

A contrast-enhanced CT scan of the neck showed an irregular exophytic enhancing lesion at the right base of the tongue, with foci of calcification and no obvious direct arterial feeding or venous drainage seen (Figure 1). However, due to limitations in assessing the depth of the lesion on the CT scan and difficulty in distinguishing between hemangioma and lymphatic malformation, we opted for contrast-enhanced MRI of the neck. The MRI revealed a well-defined lesion measuring 1.3 x 1.2 x 1.7 cm over the right base of the tongue that appeared homogeneously isointense on T1-weighted images and hyperintense on T2-weighted images. The lesion did not involve the extrinsic muscles of the tongue but was adjacent to the vallecula region. Post-contrast images (T2) showed incomplete enhancement of the lesion at the right base of the tongue, with no apparent arterial supply toward the lesion (Figure 2).

In a multidisciplinary conference with the radiology team, we concluded that the findings of incomplete enhancement and foci of calcification pointed toward a venolymphatic malformation, with lobular capillary hemangioma considered as another potential diagnosis. The patient was explained about the various treatment options; however, he opted for surgery. As the MRI findings did not show any apparent arterial supply toward the lesion, preoperative embolization was not performed.

**Figure 3:**
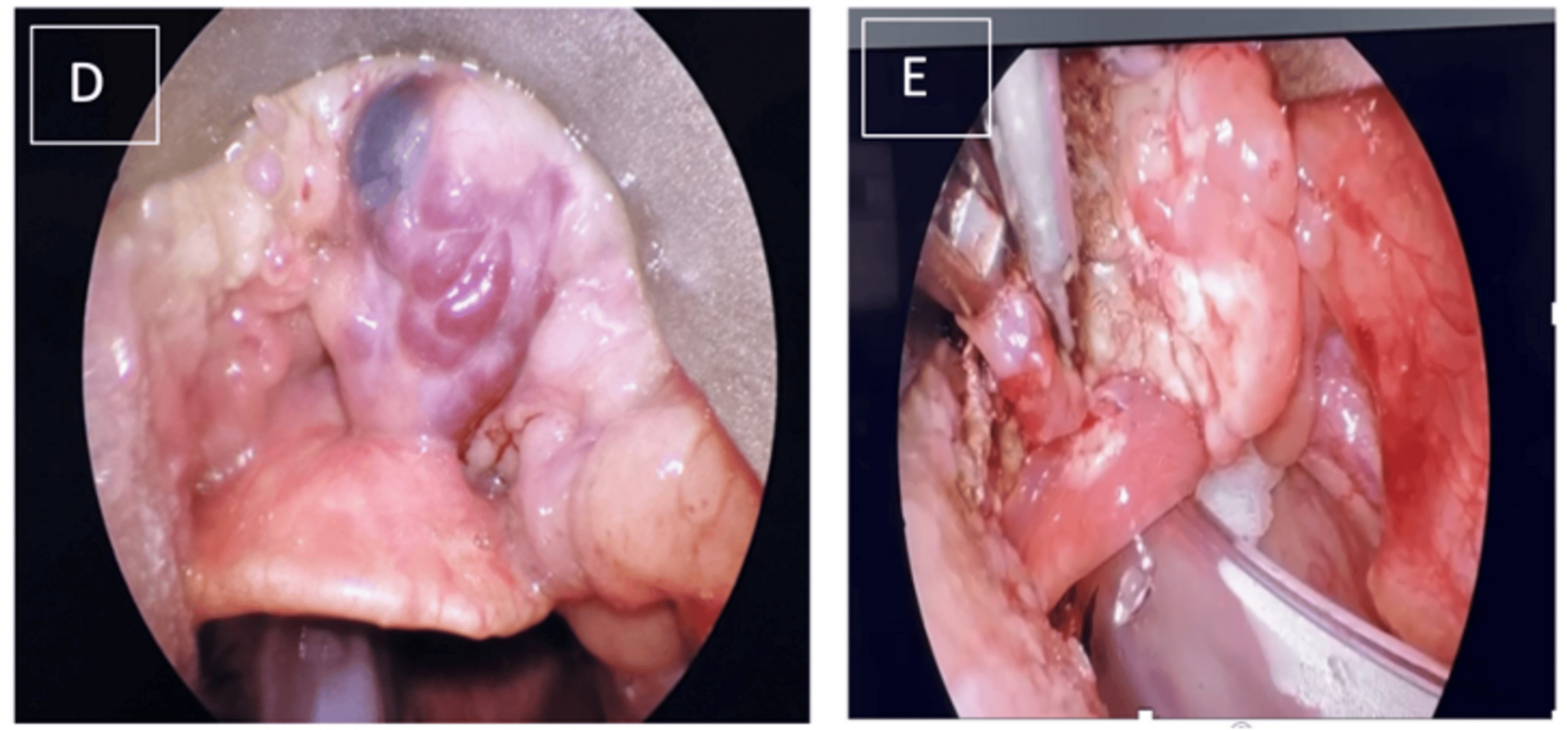
(D) A broad-based vascular tumor located at the base of the tongue, not involving the vallecula or the lingual surface of the epiglottis. (E) Excision of the vascular tumor was performed using suction diathermy and microscissors

Subsequently, he underwent direct laryngoscopy and examination under anesthesia, followed by excision of the vascular mass at the base of the tongue. Intraoperative findings confirmed the presence of a broad-based vascular tumor at the tongue base, which did not involve the vallecula or the lingual surface of the epiglottis. Excision was performed using suction diathermy and microscissors. Manipulation of the mass during surgery revealed brownish serous fluid (Figure 3). The excised tongue base mass was sent for histopathological examination, which ultimately confirmed it to be a cavernous hemangioma. Postoperatively, the patient recovered well with no signs of aspiration.

## Discussion

The terms "capillary" and "cavernous" hemangioma are considered outdated, and lesions are now more appropriately classified based on the depth of the lesion as superficial, deep, and compound hemangioma [5]. Superficial hemangiomas originate from the papillary dermis and present as bright red macular or papular masses (previously termed capillary or strawberry hemangioma). Deep hemangiomas arise from the reticular dermis or subcutaneous tissues and appear as bluish or relatively colorless masses (previously termed cavernous hemangioma) [6]. The differences between these classifications are highlighted in Table 1.

**Table 1:** Differences between cavernous, capillary, and mixed hemangioma

Clinical presentation	Cavernous hemangioma	Capillary hemangioma	Mixed hemangioma
Color and appearance	Cavernous hemangiomas often present as bluish or purple masses due to their larger, deeper blood vessels.	Capillary hemangiomas are typically bright red and have a more superficial appearance.	Mixed hemangiomas contain elements of both cavernous and capillary components, leading to a combination of the clinical features of both types.
Consistency	They are usually soft and compressible to the touch.	They may feel firm and rubbery.	
Symptoms	Patients may experience symptoms such as pain, dysphagia (difficulty swallowing), or a sensation of a lump in the throat (globus sensation).	While they can also cause symptoms like pain or difficulty swallowing, they may be more associated with bleeding or ulceration due to their superficial nature.	

Both hemangiomas and vascular malformations are endothelial malformations. Hemangiomas typically start small or are absent at birth and may go unnoticed initially by parents and caregivers. Shortly after birth, they enter a proliferative phase characterized by rapid growth that can last several months. This phase is followed by a period of stationary and subsequent involution [6]. In contrast, vascular malformations are present at birth and grow in proportion to the child's development, persisting throughout the patient’s life [6].

Cavernous hemangiomas are characterized by large vessels with occasional mitotic activity and typically have a longer clinical history. They represent approximately 5% of vascular malformations diagnosed by angiography and histology in the head and neck region. While commonly observed in individuals aged between the third and fifth decades of life, they can also occur in children and elderly patients. Remarkably, around 70% of cavernous hemangiomas resolve spontaneously by adolescence. Additionally, 50% of cases are associated with skin hemangiomas [7].

Clinically, cavernous hemangiomas are more prevalent in females, occurring twice as frequently in males. They typically present as larger lesions that are less defined and do not tend to regress. Structurally, cavernous hemangiomas consist of deep, irregular dermal channels filled with blood. These lesions are characterized by intricate networks of thin-walled cavernous vessels or sinusoids, interspersed with minimal connective tissue stroma [8].

The two noninvasive imaging techniques most useful in evaluating vascular malformations are MRI and sonography. The primary goals of imaging these vascular anomalies include characterizing the lesion and determining the anatomic extent of the disease, particularly whether adjacent vital structures such as neurovascular bundles are involved. MRI is highly valued for its superior contrast resolution, ability to assess flow dynamics, and depiction of deep and adjacent structures without ionizing radiation. For MRI, T1-weighted, fat-saturated T2-weighted, and gradient-echo (flow-weighted) MR images are preferred [6].

Features such as mass with flow voids and intermediate/hypointense signals on T1-weighted images, along with flow voids and high signals on T2-weighted images and homogenous enhancement on post-contrast enhanced images, indicate hemangiomas. In contrast, venous malformations often appear as either multiple serpentine tubular structures or amorphous dilated channels in imaging studies. They typically exhibit intermediate signals on T1-weighted images and high signals on T2-weighted images, with dynamic contrast-enhanced MRI showing delayed enhancement. The presence of phleboliths, calcified nodules within venules, veins, or sinusoidal vessels, is diagnostic of venous malformations [6]. Table 2 summarizes the MRI findings for hemangioma versus venous malformation.

**Table 2:** Summary of differences in MRI findings between hemangioma and venous malformation MRI: magnetic resonance imaging

Hemangioma	Venous malformation
Mass with flow voids and intermediate/hypointense signal on T1-weighted images, flow voids, and high signal on T2-weighted images, homogenous enhancement on contrast-enhanced images	Multiple serpentine tubular structures or amorphous dilated channels containing intermediate signal on T1-weighted images (the presence of internal hemorrhage or proteinaceous content may lend a higher signal on T1WI or fluid-fluid levels), high signal on T2-weighted images, intermediate signal on gradient echo sequences, and delayed enhancement on dynamic contrast-enhanced MRI. When present, phleboliths are diagnostic of venous elements.
Involuting hemangiomas can indicate areas of fibrofatty tissue with associated high signal intensity on T1-T1-weighted images and less contrast enhancement than that of proliferating hemangioma	Phleboliths, if present, can manifest as round, low-signal-intensity lesions in MR imaging (phleboliths small blood clots that occur in a vein, which usually hardens over time due to calcifications)
Calcified thrombi found in veins, venulae, and sinusoidal vessels are phleboliths, especially of cavernous type hemangiomas formed due to changes in blood flow	Imaging features typical of vascular malformations include their invasive nature, disregard for facial tissue boundaries, and encroachment into various tissue types like muscle and subcutaneous fat

Phleboliths, calcified thrombi occurring within venules, veins, or sinusoidal vessels, are characteristic features of venous or cavernous hemangiomas. Although hemangiomas are relatively common in the head and neck region, they rarely exhibit phleboliths in this area. Approximately 40% of venous malformations in the head and neck region may be associated with phleboliths and are usually small radiopacities, which could be occurring singly or multiple calcifications and may be round or oval, making these calcifications typical features of venous malformations [9,10]. In our case, the presence of calcifications could have led to misreporting as a venous malformation.

The treatment of hemangiomas presents a therapeutic challenge depending on their location, functional impairments, size, and bleeding risk. Asymptomatic hemangiomas can be managed conservatively. However, symptomatic lesions presenting with dysphagia, dyspnea, mass effect, or hemorrhage may require excision. Complete surgical removal offers the best chance of cure, although extensive benign lesions often necessitate significant tissue sacrifice.

Sclerotherapy is another form of conservative treatment that uses the physical, chemical, and biological properties of agents to disrupt target tissues, causing sclerosis by inducing an inflammatory response. An effective sclerosing agent such as sodium tetradecyl sulfate (STS) diffuses into target tissues and initiates sclerosis. Studies suggest that 3% STS is safe with recommended dosages between 0.5-2 ml depending on lesion size [8].

Nd:YAG laser has proven effective and safe for treating vascular lesions in the head and neck [11]. However, cases treated with CO2 laser have reported functional disabilities in swallowing, chewing, and speaking [5]. Corticosteroids have also been used to reduce the size of lesions, though intralesional injections may lead to ulceration [5]. Surgical excision remains the treatment of choice.

Propranolol has emerged as an effective treatment for infantile hemangiomas, comparable to oral steroids but without the risk of immunosuppression. Studies suggest better outcomes with propranolol compared to intralesional or topical steroids, although complications such as hypoglycemia and hypotension have been reported [12]. Recurrence following propranolol treatment has also been reported [13].

The patient was informed about various treatment options and opted for surgery due to lower recurrence rates and to alleviate obstructive symptoms. The patient recovered well, and there was no evidence of recurrence after three months of follow-up, and his symptom of globus sensation resolved.

## Conclusions

The case presented underscores the diagnostic challenges and therapeutic considerations in managing cavernous hemangiomas, particularly when located at the tongue base. Despite initial ambiguity in imaging findings, careful multidisciplinary evaluation and surgical intervention led to the definitive diagnosis and successful treatment of the vascular mass. This case highlights the importance of comprehensive imaging modalities like MRI in delineating lesion characteristics and guiding therapeutic strategies. Ultimately, surgical excision remains pivotal in achieving curative outcomes for symptomatic hemangiomas, emphasizing the need for tailored management approaches based on individual patient presentations and preferences.
